# Electronic health records in outpatient clinics: Perspectives of third year medical students

**DOI:** 10.1186/1472-6920-8-13

**Published:** 2008-03-31

**Authors:** Emran Rouf, Heidi S Chumley, Alison E Dobbie

**Affiliations:** 1 Department of Internal Medicine, University of Missouri – Kansas City School of Medicine, Kansas City, Missouri, USA; 2 Department of Family Medicine, University of Kansas School of Medicine, Kansas City, Kansas, USA; 3 Department of Family and Community Medicine, University of Texas Southwestern Medical Center, Dallas, Texas, USA

## Abstract

**Background:**

United States academic medical centers are increasingly incorporating electronic health records (EHR) into teaching settings. We report third year medical students' attitudes towards clinical learning using the electronic health record in ambulatory primary care clinics.

**Methods:**

In academic year 2005–06, 60 third year students were invited to complete a questionnaire after finishing the required Ambulatory Medicine/Family Medicine clerkship. The authors elicited themes for the questionnaire by asking a focus group of third year students how using the EHR had impacted their learning. Five themes emerged: organization of information, access to online resources, prompts from the EHR, personal performance (charting and presenting), and communication with patients and preceptors. The authors added a sixth theme: impact on student and patient follow-up. The authors created a 21-item questionnaire, based on these themes that used a 5-point Likert scale from "Strongly Agree" to "Strongly Disagree". The authors emailed an electronic survey link to each consenting student immediately following their clerkship experience in Ambulatory Medicine/Family Medicine.

**Results:**

33 of 53 consenting students (62%) returned completed questionnaires. Most students liked the EHR's ability to organize information, with 70% of students responding that essential information was easier to find electronically. Only 36% and 33% of students reported accessing online patient information or clinical guidelines more often when using the EHR than when using paper charts. Most students (72%) reported asking more history questions due to EHR prompts, and 39% ordered more clinical preventive services. Most students (69%) reported that the EHR improved their documentation. 39% of students responded that they received more feedback on their EHR notes compared to paper chart notes. Only 64% of students were satisfied with the doctor-patient communication with the EHR, and 48% stated they spent less time looking at the patient.

**Conclusion:**

Third year medical students reported generally positive attitudes towards using the EHR in the ambulatory setting. They reported receiving more feedback on their electronic charts than on paper charts. However, students reported significant concerns about the potential impact of the EHR on their ability to conduct the doctor-patient encounter.

## Background

National governmental agencies including the Institute of Medicine have called for the widespread adoption of electronic health records (EHRs) [1,2]. Evidence suggests that use of EHRs can increase delivery of preventive care, enhance monitoring of drug therapy, and improve adherence to evidence based guidelines [3-6]. In response to this call to integrate information technology into patient care, US academic medical centers are increasingly incorporating electronic health records into teaching settings [3,7,8]. As more universities adopt electronic information systems, medical students are increasingly learning to conduct and document ambulatory visits using the EHR. Medical educators are now trying to assess the impact of EHRs on medical students and post-graduate trainees. Keenan and colleagues, in a recent review article, described that residents were satisfied with electronic medical records for a number of reasons: easy access of clinical data, legibility of notes, improved problem lists and medication lists, better preventive care documentation, and reduced medical errors [9]. The authors also found that the EHR-related education included point-of-care knowledge delivery, computerized decision support systems, profiling ACGME competencies, and daily work-flow management. As for the medical students, educators are also experimenting computerized order entry, templated care notes, and virtual patients, mostly during busy clinical clerkships [9].

Despite the increasing contribution of information technology to clinical care, the literature is sparse on the impact of electronic health records on the learning process for medical students in the ambulatory setting. Of note, electronic text books, hand-held computers and personal digital assistants (PDA) are becoming commonplace in undergraduate medical education [10,11]. Medical students are using PDAs for convenient access of drug references and clinical calculators [12]. Most of them are satisfied with the use of hand-held computers and find them helpful for medical education and patient care, both in inpatient and outpatient setting [12,13]. At least one RCT has shown that the use of PDAs by medical students resulted in improved learning in evidence-based medicine through the use of PDA-based decision support software [14]. Medical educators are now developing PDA-based mechanisms to track student learning in clinical clerkships [12,15-17].

Studies with practicing physicians using the EHR have revealed concerns about adverse impacts on doctor-patient communication and patient care [18,19]. Also, observational studies of videotaped clinic encounters have raised concerns that using the EHR may adversely impact history-taking skills and doctor-patient communication [20,21]. Existing publications about medical students have largely advocated for the need for more exposure to information resources, including EHRs and computers [7,22-25]. Despite the issue's importance, a 2007 Medline search revealed no studies exploring medical students' attitudes and concerns about using EHRs in ambulatory settings for clinical learning and patient care. In our study we report third year medical students' attitudes towards clinical learning using the electronic health record in two university-based ambulatory primary care clinics.

## Methods

### Settings and subjects

The University of Kansas Medical Center is a four-year State medical school with 200 students per year and two clinical campuses. Approximately 120 third and 120 fourth year medical students train in Kansas City, the remainder train at the regional campus at Wichita. Our study subjects are 60 third year students (approximately half the Kansas City class) who completed the 12-week required Ambulatory Medicine/Family Medicine clerkship in academic year 2005–6. During the 12-week combined clerkship, all students spent 2–3 half days per week at one of two university-based primary care clinics seeing patients and documenting office visits using the Centricity^© ^EHR.

Centricity is an electronic health record solely designed for outpatient clinic visits. It has standard visit documentation interface, including templates for a variety of outpatient encounters, nursing care templates, and documents containing outpatient laboratory tests, radiologic studies, and pathologic tests. In addition, it allows providers to use age-appropriate preventive care templates and other clinical reminders for quality of care processes. We also developed a diabetes disease management template which was available with the outpatient office note template. It is also pre-loaded with drug interaction and patient education databases. By and large, both physicians and students were using the basic features of the EHR without any clinical decision support tools, automated pop-up reminder or alerts, except for the drug interaction alerts. Clinical providers, including medical students had similar access to all the basic features. However, medical students did not see the automated drug interaction feature as a prompt, which occurs right before signing prescriptions. Without the prescription signing authority, the only way for students to check drug interactions was to actually click the 'check interactions" button within the electronic note. As for clinical resources, the Centricity has accessible links to use internet and "Up-to-Date", an evidence-based clinical resource for clinicians. During a typical clinic visit, a nurse would enter vital signs, the doctor or student would then start seeing the patient in the room interacting with Centricity from a desktop computer or tablet PC. All students at the Family Medicine clinic had access to tablet PCs while students at the Ambulatory Medicine clinic used desk-top PCs for using Centricity and related patient care processes. As part of the EHR implementation process, faculty and clerkship students completed mandatory training on how to use different features of the EHR.

From a patient care perspective, the students could use the EHR's visit templates that had visit-specific history and examination fields. Thus, they could complete visit notes without remembering all the elements of history an examination items. They could also review vital signs for any visit, previous office notes and laboratory tests for their patients. They were trained to use age-appropriate health maintenance templates, online resources, and patient education materials, as appropriate for the visit type. Students working at the Ambulatory Medicine clinic also had access to the paper charts to review patient data from hospital admissions and sub-specialty clinic visits. The students had the opportunity to discuss patients' clinical presentations with their attendings, who then would teach clinical matters, as appropriate, and review student notes. The attendings were responsible for editing and signing the electronic student notes. They were encouraged to forward all notes to the students' inboxes within the EHR as a way of providing feedback on their notes. All students were also exposed to clinic settings without EHRs, for example, at the offices of their community preceptors. Prior to this study, no students in our combined Ambulatory Medicine/Family Medicine clerkship had previously used an EHR for clinical reasoning and medical decision-making during real-time patient care.

### The educational intervention

Of 60 third year medical students who completed the 12-week Ambulatory Medicine/Family Medicine clerkship between October, 2005 and February 2006, 53 (88%) consented to be surveyed regarding the impact of electronic health records on their learning in the ambulatory care setting. The study was approved as exempt by our institution's Human Subjects Committee.

### Survey design

In July 2005, we conducted a focus group of third year students to elicit their perceptions about the use of EHR during their Ambulatory Medicine/Family Medicine clerkships. The main goal of conducting the focus group was to determine a set of important concepts and themes pertinent to the use of an EHR in clinic and its impact on clinical care processes and student learning. We hoped that the themes from the focus group would help develop the study questionnaire. Five themes emerged: organization of information, access to online resources, prompts and templates from the electronic health record, personal performance (charting and presenting), and communication with patients and preceptors. We added a sixth theme: impact on student and patient follow-up. We wrote 3–6 stems for each of these six constructs to create a 21-item questionnaire. The responses used a 5-point Likert scale from "Strongly Agree" to "Strongly Disagree". The instrument also included 2 open-ended questions at the end, and one of which specifically asked students to comment on learning in an EHR-enabled setting, compared to an office setting with paper charts.

### Survey administration

We assembled the questionnaire using Survey Monkey^© ^and emailed an electronic link to each consenting student immediately following their clerkship experience in Family Medicine. We re-sent the survey to non-responders 1–2 weeks later.

## Results

33 of 53 consenting students (62%) returned completed questionnaires. Most students liked the EHR's ability to organize information, with 70% of students responding that essential information was easier to find electronically. However, the EHR did not encourage most students to use online resources beyond their reported baseline levels. Only 33% and 36% of students reported accessing online patient information or clinical guidelines more often when using the EHR than when using paper charts. Most students (72%) reported asking more history questions due to EHR prompts, and 39% ordered more clinical preventive services. Most students (69%) reported that the EHR improved their documentation, but only 24% thought it improved their case presentations. 39% of students responded that they received more feedback on their EHR notes compared to paper chart notes. Only 64% of students were satisfied with the doctor-patient communication with the EHR, and 48% stated they spent less time looking at the patient (For full results, see Table 1).

**Table 1:** Percentage of students who answered strongly agree/agree, neutral, and disagree/strongly disagree for statements comparing an EHR to a paper chart

**Construct**	**Items**	**SA/A %**	**N** %	**D/SD**
Organization of information	It was easier to find essential information in the EHR	70	15	15
	I prefer looking for patient information in the EHR	67	18	15
	I prefer the layout/organization of the EHR	57	21	21
Access to online resources	I accessed online clinical guidelines more often when using an EHR	36	27	36
	I accessed online information for patients more often when using an EHR	33	27	39
	I accessed online information about medications more often when using an EHR	15	30	54
Prompts from the EHR	I was prompted to ask more history questions	72	9	18
	I was prompted to order more clinical preventive services	39	33	27
	I learned about medication interactions from using the EHR	30	18	51
Personal Performance	My documentation was more complete in the EHR	69	12	18
	The normal exam defaults in the EHR helped me find the words I needed to write my notes	63	15	21
	My presentations were better organized when I used an EHR	24	30	45
Follow-up	I accessed patients' tests results more often when using an EHR	54	21	24
	I received more feedback on my EHR notes	39	24	36
	I sent reminders to myself through the EHR to follow-up on patients	15	18	67
Patient-student- physician communication	Overall, I was satisfied with the doctor-patient communication with the EHR	64	18	18
	I spent less time looking at the patient because of the EHR	48	6	45
	I spent less time talking to the patient	24	21	54
	The EHR improved my ability to establish rapport with patients	24	39	38
	My patients liked that I was using an EHR	21	76	3
	The EHR adversely affected communication with my preceptor	9	15	76

EHR = Electronic Health Record; SA = Strongly Agree; A = Agree

There were too few written comments for a thematic analysis; however several students commented on the ease of documentation and accessibility of the patient chart when using the EHR. Table 2 outlines selected student comments regarding the use of EHR. Students valued learning to use an EHR – "it's important to have an exposure to an EHR as a student because it will be widely used in the future." They also had concerns about patient care with EHR (Table 2) – "more challenging to talk to the patient and type at the same time", and "the only concern is when it (the EHR) doesn't work it becomes paralyzing."

**Table 2:** Selected student comments

1	"It's important to have exposure to an EHR as a student because it will be widely used in the future."
2	"Information collected was more complete"
3	"Using an EHR was helpful in terms of learning the appropriate "doctor terminology" for normal and abnormal findings..."
4	"...more challenging to talk to the patient and type at the same time..."
5	"I don't like not to be able to keep good eye contact."
6	"The only concern is when it does not work, it becomes paralyzing."
7	"You have to be able to effectively work with a computer as well as learn how to efficiently use the EHR."

Question #1: During your third year, you have been in office settings with electronic and paper documentation systems. What is different about learning in settings with an EHR?

Question #2: The EHR provides clinical information about appropriate preventive care services and medication interactions. What, if any, clinical information did you learn from the EHR?

## Discussion

Our study provides interesting perspectives on how medical students in our institution viewed the impact of the EHR on their learning in an ambulatory clinical setting. While most students preferred the EHR as an organizational aid for asking more history questions and for better documentation of visit notes, it was clear that most were not utilizing key features of the EHR to augment their learning. Our students reported not using online resources (e.g., "Up-to-Date", patient education materials) and medication interactions more often.

A number of factors may explain these findings. Most students are of a generation that is familiar with computers and are able to type well. Therefore, it is not surprising that they prefer computer-based documentation, and overall, preferred EHR to a paper chart. However, despite having an adequate training, twelve weeks might have been insufficient for many students to learn how to use the EHR to its full potential, including linking to on-line resources and using preventive care templates and medication interactions. Arguably, the low reported use of such resources may simply reflect a developmental learning process. Perhaps, a number of factors, such as the clinical work-flows, design of and training with an EHR influence how users perceive the utility and benefits of an EHR. Of note, the relevant literature reveals that there is no correlation between the length of EHR experience and use of it's features. Arguably, student learning and perceptions could have been different if we used a different electronic record. We believe our EHR, like others out in the market, offered a similar set of features for outpatient clinical practice. Furthermore, we do not know if students' perceptions were influenced by the attendings' EHR use. We do not have any knowledge about the extent to which attendings were using the EHR and encouraging students to use those features during clinical encounters. We were interested to explore the extent to which medical students, who are typically expected to do detailed history and examination, view EHR-related tools as facilitating to learning and patient care.

Students reacted differently to different prompts from the medical record. Most reported that the prompts made them ask more history questions, but we do not know if this increased questioning resulted in a more effective clinical encounter. Although only around 40% reported ordering more preventive services when prompted by the EHR, that 40% translates into a great many preventive services that students might not otherwise have ordered. Similarly, only 30% reported learning about medication interactions from the EHR prompts, and it is not clear whether students already knew about these drug interactions or if they ignored the prompts. Also, since the nursing staff in our clinic updates a patient's medication list before he/she sees the provider, students may not have viewed as many medication interaction prompts as if they had updated the medication lists personally.

Although most students reported that their documentation was better and more complete with the EHR, only 24% reported that their oral presentations were better organized. The reasons for this are unclear. Our cohort were mid year third year students, so many may have considered that their baseline presentations were already well-organized. Also, these are self-reported data, and many students may not have been able to accurately judge the extent of any improvement on their presentations.

Regarding the impact of EHR use on patient follow up, over 50% of students reported accessing patients' test results electronically more often than from a patient's paper chart. This evidence of increased patient follow up is encouraging, but prompts questions about how frequently (or infrequently) do students routinely follow up on ambulatory labs they have ordered from paper charts? In a related point, only 15% of students used a very simple electronic prompt to remind themselves to follow up on patients. Either students were using other prompts (e.g. a notebook), simply remembering, or not following up at all.

In concordance with the existing literature, the students in our study raised concerns about how an EHR can impact patient, student and physician communication. While 64% reported overall satisfaction with doctor-patient communication with the EHR, many felt that the EHR might be a barrier for relationship building tasks (talking to, looking at, and building rapport with patients) during clinical encounters. Almost half (48%) reported spending less time looking at patients because of the EHR, and 34% reported spending less time talking to patients. Only 24% agreed or strongly agreed that using the EHR improved their rapport with the patient, and only 21% agreed or strongly agreed that their patients liked them using the EHR. More advanced learners (e.g., internal medicine residents) raised similar concerns in a study done at a VA (Veterans Affairs) primary care clinic. In that study, resident physicians and their patients were more concerned about the interpersonal aspects of care in the presence of an exam room computer, compared to faculty physicians and their patients [26]. It is possible that increasing experience with EHRs along with overall clinical experience may positively impact a provider's attitudes to the effect of the EHR on the doctor-patient relationship.

Students reported some impact of the EHR on the preceptor-learner relationship. Fortunately, only 9% of students considered that precepting with the EHR adversely impacted communication with their teachers. In contrast, almost 40% reported receiving more feedback on their electronic charts than on their paper charts. Considering the well-documented paucity of feedback in the ambulatory setting, we consider 40% of students reporting increased feedback to be an extremely positive finding.

Our study has several limitations. We had a small sample size – one group of third year medical students from a single institution, and our response rate of 62% is lower than the 70% generally accepted for generalizability. In addition, we distributed our questionnaire electronically, which may have biased our respondents towards those who are more comfortable with electronic technology. If that bias did exist, we might postulate that non-responding students would be less comfortable with the EHR, use its features less, and be more concerned about its impact on the doctor-patient relationship. We did not use an existing survey instrument, but we believe it had good content and face validity, as it was assembled by an expert panel from combined personal experience, recent literature findings and student-generated themes. However, we conducted no further formal validation on the instrument. Although, we added two open-ended questions in the survey instrument, we could have asked more specific questions to elicit finer details of responses not captured in the questionnaire (for example, "what features of an EHR would you want in your clinical practice?"). Our results are based on students' self-report, and as such, the data need to be interpreted carefully. We did not videotape any clinical encounters of our students nor did we query the EHR to obtain actual usage data, both of which could have yielded different and meaningful findings. We also wonder if some technological enhancements in our EHR, such as direct order-entry, pop-up reminders, easy-to use decision support tools, and use of tablet PCs could provide different results. Last but not the least, our lack of knowledge about attending physicians' behaviours and role modelling in an EHR-enabled setting is worth mentioning. Students' opinions in this regard may largely be dictated by whether or not they worked with technologically-savvy physicians. Despite these limitations, we believe our study should be viewed as a small but important pilot experiment. It adds to the paucity of literature on this extremely important subject, and raises several important questions to be addressed in future studies.

## Conclusion

In our setting, third year medical students reported generally positive attitudes towards using the EHR in the ambulatory learning environment, although they consistently underused many technical features of the record (e.g. the ability to check medication interactions). They reported performing more complete histories and documentation. They reported receiving significantly more feedback from their preceptors on their electronic charts than on paper charts. However, students reported significant concerns about the potential impact of the EHR on their ability to conduct the doctor-patient encounter.

Since all current medical students are highly likely to use EHRs in their future practices, medical educators should attend to the 'red flags' raised by our study. As a community of educational scholars, we must conduct more research into how to best integrate the EHR into students' clinical learning. We must investigate why students consistently under-use important features such as medication interactions, health maintenance prompts and on-line resources such as clinical guidelines. We must also study the impact of the EHR on the teacher-learner relationship. There will likely be other benefits or drawbacks to precepting using the EHR apart from a reported increase in feedback. Finally, we must address learners' concerns about the impact of the EHR on the doctor-patient relationship. Perhaps Deans and clinical skills course directors should consider incorporating computers into basic communications skills classes for first and second year students.

## Competing interests

The author(s) declare that they have no competing interests.

## Authors' contributions

ER participated in the design and coordination of the study and helped to draft the manuscript. HC drafted the human subjects' protocol, participated in the design of the survey and helped to draft the manuscript. AD participated in the design of the study and the survey and helped to draft the manuscript. All authors read and approved the final manuscript.

## Pre-publication history

The pre-publication history for this paper can be accessed here:


